# Rab GTPases and the Autophagy Pathway: Bacterial Targets for a Suitable Biogenesis and Trafficking of Their Own Vacuoles

**DOI:** 10.3390/cells5010011

**Published:** 2016-03-08

**Authors:** María Milagros López de Armentia, Celina Amaya, María Isabel Colombo

**Affiliations:** Laboratorio de Biología Celular y Molecular, Instituto de Histología y Embriología (IHEM)-CONICET, Facultad de Ciencias Médicas, Universidad Nacional de Cuyo, Casilla de Correo 56, Centro Universitario, Parque General San Martín, 5500 Mendoza, Argentina; milagrosarmentia@gmail.com (M.M.L.A.); celinaamaya@yahoo.com.ar (C.A.)

**Keywords:** autophagy, Rab GTPases, bacterial pathogens, intracellular bacteria

## Abstract

Autophagy is an intracellular process that comprises degradation of damaged organelles, protein aggregates and intracellular pathogens, having an important role in controlling the fate of invading microorganisms. Intracellular pathogens are internalized by professional and non-professional phagocytes, localizing in compartments called phagosomes. To degrade the internalized microorganism, the microbial phagosome matures by fusion events with early and late endosomal compartments and lysosomes, a process that is regulated by Rab GTPases. Interestingly, in order to survive and replicate in the phagosome, some pathogens employ different strategies to manipulate vesicular traffic, inhibiting phagolysosomal biogenesis (e.g., *Staphylococcus aureus* and *Mycobacterium tuberculosis*) or surviving in acidic compartments and forming replicative vacuoles (e.g., *Coxiella* *burnetti* and *Legionella pneumophila*). The bacteria described in this review often use secretion systems to control the host’s response and thus disseminate. To date, eight types of secretion systems (Type I to Type VIII) are known. Some of these systems are used by bacteria to translocate pathogenic proteins into the host cell and regulate replicative vacuole formation, apoptosis, cytokine responses, and autophagy. Herein, we have focused on how bacteria manipulate small Rab GTPases to control many of these processes. The growing knowledge in this field may facilitate the development of new treatments or contribute to the prevention of these types of bacterial infections.

## 1. Introduction

### Interaction of Rabs, Autophagy and Intracellular Bacteria

Rab (Ras-related proteins in brain) GTPases are a family of proteins encoded by a group of at least 60 genes. This must be considered as a minimum estimate, as a small part of the genome still remains to be sequence [1]. Rab GTPases cycle between active and inactive states, GTP-bound or GDP-bound forms, respectively. In the active conformation, Rabs interact with a high variety of Rab-effector proteins to participate in membrane trafficking and intracellular signaling [2,3,4]. To date, 70 distinct Rab proteins have been identified, and each one is believed to be specifically associated with a particular organelle or pathway. Rabs are present in all intracellular membrane compartments (endoplasmic reticulum, Golgi, endosomes and lysosomes), at the plasma membrane, the nucleus [5] and the mitochondria [6]. However, they are best known for their crucial roles in the endocytic and the exocytic pathways, in the regulated secretory paths where they control anterograde and retrograde trafficking between compartments to manage cargo delivery and membrane recycling [6,7].

Autophagy is an intracellular process that involves degradation of damaged organelles, protein aggregates and intracellular pathogens with a key role not only in maintaining cellular homeostasis and guaranteeing adequate energy levels, but also as a defense mechanism against microorganisms, a process specifically known as xenophagy [8,9].

Autophagy process involves the enclosure of cytoplasmic material by an isolation membrane, called phagophore, which extends to form a double-membrane structure, the autophagosome that finally fuses with lysosomes to degrade the trapped material [10,11,12]. Autophagosome formation is regulated by at least 36 autophagy-related genes (Atg). The Atg1-UNC-51-like kinase (ULK) complex triggers autophagy, then the PI3 (class III phosphatidylinositol 3) kinase complex produces PI3P (essential component of autophagosome formation). Finally, the Atg12–Atg5–Atg16L1 ubiquitin-like conjugation system, that mediates the elongation of the membrane and the conversion of LC3 I (the mammalian orthologue of the yeast Atg8) to its phosphatidylethanolamine-conjugated LC3-II form, participates in the closure of the phagophore to form the autophagosome [13].

Intracellular pathogens are internalized by professional phagocytes (macrophages, dendritic cells) or non-professional phagocytes (epithelial cells) and localize into a vacuole, called phagosome. The microbial phagosome matures by fusion events with early and late endosomal compartments and lysosomes, which are controlled by Rab GTPases, to degrade the internalized microorganism [14,15,16]. Interestingly, in order to survive in the phagosome and replicate, pathogens employ a wide variety of strategies to manipulate vesicular traffic, some can inhibit phagolysosomal biogenesis (e.g., *Staphylococcus aureus or Mycobacterium tuberculosis*) or survive in acidic compartments forming replicative vacuoles (e.g., *Coxiella burnetti or Legionella pneumophila*) [17,18,19,20].

## 2. Intracellular Bacterial Pathogens: Interplay with Rabs and Autophagy

### 2.1. Mycobacterium Tuberculosis

**Figure 1:**
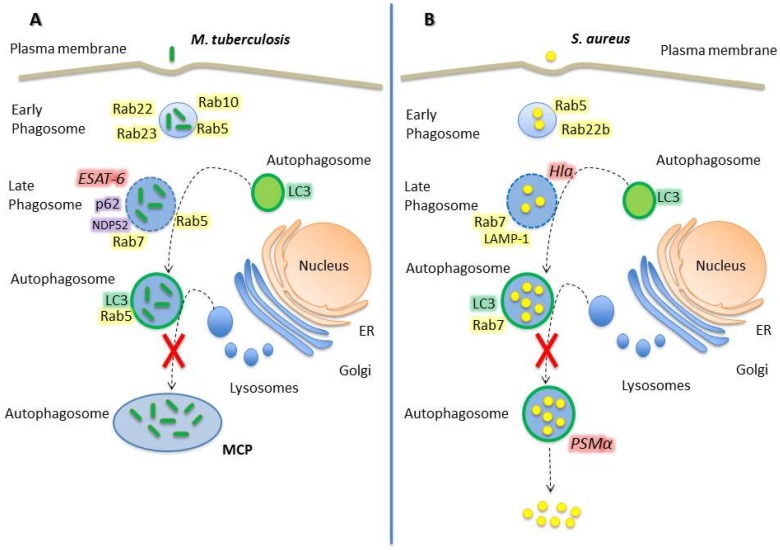
Rab GTPases and vesicular trafficking modulation by *Mycobacterium tuberculosis* and *Staphylococcus aureus*. (**A**) *M. tuberculosis* initially resides in a phagosome characterized by the presence of Rab5, Rab10, Rab22b and Rab23 that matures to a late compartment. Approximately 1 h p.i. *Mtb* secretes ESAT-6, a pore forming toxin, and lyses the membrane promoting p62 and NDP52 anchorage. Together, these proteins stimulate the autophagic pathway, and the damage phagosome is entrapped by an autophagosome. Rab5 remains in the phagosome, inhibiting the proper maturation of the compartment and as a consequence, it inhibits the lysosomal fusion. Finally, *Mtb* replicates inside the mycobacterial-containing phagosome (MCP). (**B**) *S. aureus* transits through an early phagosome with Rab5 and Rab22b that quickly maturates (15 min p.i.) to a late compartment marked by Rab7 and LAMP-1. Alpha hemolysin (Hla) is secreted by the bacteria and causes membrane damage. Autophagy is stimulated by the toxin and autophagosomes are recruited to the damage phagosome. *Sa* replicates inside the autophagosomes that does not mature to autophagolysosome due to the inhibition of lysosomal fusion. Then, phenol soluble modulin alpha (PMSα) mediates *Sa* escape to the cytoplasm, where bacteria continue replicating.

*Mycobacterium tuberculosis* is the causative agent of tuberculosis (TB) in humans. This microorganism employs a wide array of immune modulators to invade and proliferate in professional phagocytic cells, such as macrophages, neutrophils, monocytes, and dendritic cells, inhibiting the acidification of the phagosome and the recruitment of lysosomal enzymes [21,22]. Rab GTPases play a key role in *M. tuberculosis* (*Mtb*) infection. *Mtb* modifies the recruitment of Rab proteins to the phagosomal membrane, altering the signals necessary for proper maturation and late fusion with lysosomes (Figure 1).

Once *Mtb* is phagocytosed, it resides in an early compartment characterized by the presence of different Rab-GTPases, which transiently associate to the membrane to allow phagosomal maturation. After 10 min of infection, Rab34 and Rab5 are recruited in high percentage (~85%) whereas Rab22 and Rab23 in low percentage (~30%). Rab22 and Rab23 are specific to early endosomes/phagosomes and, after 1 h post-infection (p.i.), the recruitment to *Mtb* phagosomes decreases to 10%, indicating maturation of the compartment [23]. Rab22a localizes to early endosomes, plasma membrane and recycling pathways and, as mentioned above, it is recruited to *Mtb* phagosomal membrane at early stages (*i.e.*, 10 min p.i.) in a high proportion, and decreases to less than 10% after 60 min p.i. [4,23]. However, Rab22a has been recognized as a key Rab GTPase in *Mtb* phagosome maturation; its role being evidenced by the conversion of mycobacterial phagosomes into a Rab7 positive vacuole upon Rab22a knockdown. The latter phenomenon suggests that Rab22a confers signals to prevent the acquisition of the late endosomal marker Rab7 and inhibits maturation into a late endosomal/lysosomal compartment [24]. Hence, *Mtb* actively recruits and maintains Rab5 and Rab22a on its phagosome, thus avoiding Rab7 acquisition and preventing phagolysosome biogenesis.

Rab5 is associated with phagosomes immediately after phagocytosis and facilitates the recruitment of Rab5 effector proteins such as EEA1 (early endosomal autoantigen 1) and class III phosphatidylinositol-3-phosphate kinase (PI3K) [25]. In *Mtb* containing-phagosomes, Rab5 is recruited at early stages and its maintenance over time causes maturation disruption due to an inhibition of the Rab5–Rab7 switching. EEA1 is crucial for phagosomal maturation, and its recruitment to *Mtb* phagosomes is altered as a part of the mycobacterial phagosome maturation blockade. LAM, the heavily glycosylated mycobacterial phosphatidylinositol analogue, inhibits EEA1 recruitment to phagosomal membranes, which causes a disruption in the delivery of lysosomal hydrolases and V_o_ H^+^ ATPase from the Trans-Golgi network (TGN) to the phagosome [26,27].

Indeed, tuberculosis bacilli inhibits fusion of lysosomes with phagosomes through the selective exclusion of the GTPase Rab7 and lysosomal-associated membrane protein 1 (LAMP1) coupled with the retention of Rab5 on the phagosome [28]. Seto *et al.* have shown that Rab7 recruitment to *Mtb* phagosome decreases in a time-dependent manner, starting at 30 min p.i. at high levels (~80%) and decreasing after 180 min to approximately 30%. Cumulative data suggest that mycobacterial proteins and phagosomal membrane lipids may reduce Rab5-Rab7 conversion, specifically blocking the recruitment of RILP (Rab interacting lysosomal protein) to Rab7, or mycobacterial proteins may act as Rab7-GAPs to inactivate Rab7 and inhibit interaction with RILP [29]. These alterations in phagosomal maturation are key features to allow *Mtb* persistence and proliferation.

Recently, Cardoso *et al.* have demonstrated that Rab10, a Rab localized predominantly in early endosomes and the Golgi, is also recruited to *Mtb* phagosomes and regulates the transition of the nascent phagosome to an early phagosome. Hence, Rab10 is acquired even before Rab5, acting upstream and modulating the maturation of *Mycobacterium*-containing phagosomes. Indeed, the overexpression of the constitutively active mutant of Rab10 rescued the maturation of live-*Mycobacterium*-containing phagosomes [30].

*Mtb* secretes multiple virulence factors via the general Sec and Tat pathways, and via specialized ESX secretion systems, also called type VII secretion systems. The ESX-1 secretion system is an important virulence factor which secretes the 6 kDa early secreted antigenic target (ESAT-6) and its protein partner, the 10 kDa culture filtrate protein (CFP-10). Both ESAT-6 and CFP-10 are exported as a heterodimer through the secretion system [31,32]. It has recently been shown that ESAT-6 inserts into membranes and forms a membrane-spanning pore that damages the phagosomal membrane [33]. The damage generated in the phagosomal membrane is a signal for autophagy induction. Adaptor proteins, such as NDP52 and p62, recognize the damage and stimulate LC3 binding to phagosome membrane to capture *Mtb* in an autophagosome [34]. Interestingly, *Mtb* can survive in the autophagosome due to its ability to interrupt the autophagic flux, by avoiding Rab7 recruitment to form the amphisomes and, as a consequence, interfering in autophagolysosome biogenesis [35]. Of note, nutritional starvation has been demonstrated to promote the binding of LC3-II to the phagosomal membrane and the maturation and acidification of *Mycobacterium* phagosomes by increasing the recruitment of late endosomal/phagosomal markers (e.g., LAMP-1 and cathepsin D) directly affecting *Mycobacterium* survival [36]. When autophagy is induced, it acts as a host’s defense mechanism against *Mtb,* stimulating its lysosomal degradation. In contrast, when autophagy is not activated by starvation or other stimuli, *Mtb* is capable of surviving inside autophagosomes by inhibiting lysosomal fusion and, as a consequence, autophagolysosome biogenesis [34]. Moreover, Castillo *et al.* demonstrated that the *in vivo* role of autophagy against tuberculosis is both antibacterial and anti-inflammatory. Specifically, conditional gene-knockout mouse Atg5^fl/fl^ LysM-Cre+ (Atg5 deletion on myeloid linage) infected with *Mtb* showed increased infection and high pulmonary inflammation, characterized by neutrophil infiltration and IL-17 response with augmented IL-1α levels. Thus, autophagy acts *in vivo* suppressing bacilli growth and protecting against tissue necrosis and lung pathology [37]. Indeed, a recent work showed that Atg5 plays a unique role in protection against *Mtb* by preventing polymorphonuclear-mediated immunopathology. Additionally, loss of Atg5 in polymorphonuclear cells can sensitize mice to *Mtb* infection [38].

Rab8b has previously been shown to be recruited to *Mtb* phagosomes at late infection stages; however, its role in *Mtb* infection was unknown [23]. Recently, Pilli *et al.* have shown that Rab8b is involved in the autophagy clearance of *Mtb var. bovis BCG.* In addition, its downstream interacting partner, the innate immunity regulator TBK-1, is necessary for the autophagic maturation. TBK-1 phosphorylates the autophagic adaptor p62 on Ser-403, a residue required for the autophagic function of p62 [39,40].

Mycobacterial phagosome maturation is closely related to the autophagic pathway, but *Mtb*-containing autophagosomes do not mature into autolysosomes. In conclusion, *Mtb* takes advantage of Rab GTPases and of autophagy to establish a perfect replicative niche, guaranteeing survival.

### 2.2. Staphylococcus Aureus

*Staphylococcus aureus* causes a wide range of diseases in humans, from local infections (e.g., dermatitis, folliculitis, and abscesses) to life-threatening systemic infections (e.g., endocarditis, pneumonia, atherosclerosis, and septicemia). As a rule, *S. aureus (Sa)* has been considered an extracellular pathogen, but increasing evidence indicates that this bacterium can invade cells and replicate intracellularly, leading to staphylococcal persistence and chronic disease [41,42,43,44]. *Sa* initially resides in a phagosome and replicates in this compartment by inhibiting lysosomal degradation, which is accomplished by the secretion of toxins [45,46].

Rab GTPases are essential in *Sa* infection, participating in both phagosome formation and maturation. At the early stage of infection (10 min approximately), the *Sa*-containing phagosome acquires different markers, such as Rab5 and Rab22b at a percentage of 30% and 90% of the phagosomes, respectively. Subsequently, these Rab GTPases decrease considerably and Rab8, Rab11, Rab20, Rab22a, Rab32 and Rab38 are recruited transiently, peaking at 30 min and/or 1 h p.i. [23]. Even though the specific role of Rab22b in *Sa* phagosome is not clear, it is well known that this molecule functions in a vesicular transport route from the Trans-Golgi Network (TGN) to the early endosomes [47]. It has recently been shown that Rab5 plays an important role in *Sa* infection, as demonstrated by the reduced susceptibility to infection observed in cell knockdown for Rab5 [48], which likely affects bacteria internalization.

The Rab6 GTPase plays important roles in the transit of proteins through the Golgi complex, regulating the transport between Golgi, endoplasmic reticulum, plasma membrane and endosomes [49,50,51]. A recent study has revealed that Rab6 is involved in the acidification of phagosomes and that Rab6 mediates the phagocytosis of *Sa* affecting its proliferation, since silencing of Rab6 expression caused an important decrease in the clearance of live *Sa* by RAW 264.7 cells [52].

Rab7 is recruited to 40% of *Sa*-phagosomes at 10 min p.i. whereas at 30 min p.i., the proportion of Rab7-positive phagosomes is higher than 80% and remains at this level until 6 h p.i. Localization of Rab9, Rab34 and Rab39 has shown a similar kinetics pattern to Rab7 [23]. Remarkably, *Sa*-phagosomes recruit Rab7 and LAMP-1, late compartment markers, but there is a blockade in the fusion with the lysosomal degradative compartment evinced by the absence of acidic or degradative probes, such as LysoTraker and DQ-BSA [53,54,55].

The Type VII secretion system, also termed Ess or ESX, is a key virulence determinant in *Sa*; however, the exact function of any of the Ess substrate proteins remains to be elucidated [56]. The locus on the *Sa* chromosome that controls the synthesis of a number of exoproteins has been vastly studied. This locus, which has been named *agr* for “accessory gene regulator”, is a trans-activator of a series of exoprotein genes. One important exoprotein that is controlled by the *Sa* *agr* system is the α-hemolysin (α-toxin, Hla), which is a pore-forming toxin involved in membrane damage. Alpha hemolysin is secreted as a 34 kDa soluble monomer that functions as a homo-heptameric pore-forming toxin. Previous works carried out in our laboratory have demonstrated that the addition of the purified Hla toxin to culture cells activates the autophagy pathway, evidenced by the accumulation of a large number of vesicular structures decorated with the protein LC3 [55]. In addition, we have shown that wild type *Sa* induces recruitment of LC3 to the phagosomal membrane, but the Hla-deficient strain (*Sa* 8325-4 Hla(-)) does not, indicating that the toxin secreted by the intracellular bacteria is also capable of inducing autophagy [55].

Interestingly, *Sa* subverts autophagy and replicates inside the autophagosome to finally escape to the cytoplasm, thus preventing lysosomal degradation [57]. *Sa* inhibits fusion of phagosomes with lysosomes by a mechanism dependent on *Staphylococcus*-secreted toxins. The leaky phagosomes undergo autophagy and *Sa* replicates within autophagosomes [57]. Eventually, *Sa* escapes to the host cell cytoplasm by a process that is dependent on a phenol-soluble modulin (PSMα), a protein that has cytolytic activity and belongs to the family of toxins that are soluble in phenols [58].

The regulation of the classical autophagy pathway involves a variety of proteins. One of these proteins is the serine/threonine kinase mTOR (mammalian target of rapamycin), which inhibits autophagy [59,60]. On the other hand, autophagy is regulated by the Class III phosphatidylinositol-3-kinase (PI3K), which activates the autophagic pathway. Class III PI3K, and its human orthologue hVps34, interact with Beclin-1 and p150 myristoylated kinase, activating some of the Atg proteins involved in the autophagic pathway [61]. Of note, the *Sa*-activated autophagy occurs through a PI3K/Beclin-1 independent pathway, indicating that the activation is through a non-canonical mechanism. Indeed, we have shown that cAMP is able to inhibit Hla-induced autophagy and that PKA, the classical cAMP effector, does not participate in this regulation. Furthermore, we have shown that EPAC and its effector Rap2b are involved in the regulation of Hla-induced autophagy [62].

*Sa* takes advantage of intracellular trafficking, modulating Rab GTPases recruitment to the phagosome and activating autophagy. These processes are accomplished, at least in part, through membrane damage, creating a replicative niche and avoiding degradation.

### 2.3. Coxiella Burnetii

As mentioned above, the autophagic pathway has been revealed as a component of the innate immune response against intracellular microorganisms. Nevertheless, pathogens like *Coxiella* *burnetii* (hereafter *Coxiella*) benefit from this cellular response and subvert the autophagy process resulting in a more efficient replication [63].

*Coxiella* is an obligate intracellular bacterium which is the causative agent of Q fever in humans and of coxiellosis in animals. This microorganism has developed strategies to survive in the harshest of the intracellular compartments: the phagolysosome. After internalization, the budding *Coxiella* phagosome eventually develops into a great and spacious parasitophorous vacuole (PV) that acquires lysosomal features such as acidic pH, acid hydrolases and cationic peptides, which are defense barriers aimed at clearing infectious agents from the host [64].

*Coxiella* actively contributes to the biogenesis of its PV by producing proteins that mediate phagosome stalling, autophagic interactions, and expansion and maintenance of the mature vacuole. Among the potential mechanisms facilitating these processes, *Coxiella* has the Dot/Icm type IV secretion system (T4SS). This system delivers bacterial effector proteins to the host cytosol, where they alter cellular processes to benefit the pathogen [64,65,66,67,68]. Specifically, the *Coxiella* T4SS translocates effectors when the pathogen becomes metabolically active in an acidified vacuole. The function of the T4SS is essential for intracellular replication of *Coxiella,* since mutants defective in this virulence factor are incapable of growing efficiently in the intracellular environment [69,70].

**Figure 2:**
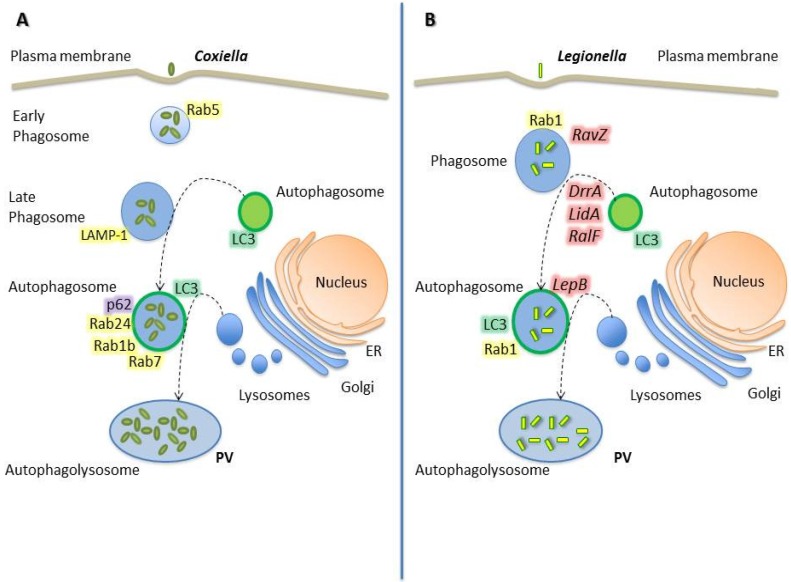
Intracellular trafficking route of *Coxiella* and *Legionella*. (**A**) *Coxiella* initially resides in a parasitophorous vacuole (PV) that takes hours to days to mature into a compartment resembling a lysosome. Rab5 and Rab7 have been shown to be sequentially present on the PV, with Rab5 showing maximal recruitment approximately 20 min p.i. before being replaced by Rab7 which remains on the PV throughout the infection. Between 6 and 12 h post- infection, the PV acquires autophagy markers including LC3 and Rab24. Then, p62 colocalizes with LC3 on PV membranes containing ubiquitinated proteins without affecting the bacterial replication. In addition, Rab1b labels the PV membrane at later p.i. times (6 h). (**B**) *Legionella* actively evades endocytic trafficking and creates an ER-derived niche. Effector proteins such as DrrA/SidM, LidA, and RalF, secreted by the T4SS, prolong the association with the ER and inhibit the immediate delivery to lysosomes. Likewise, RavZ cleaves the membrane-conjugated LC3 from pre-autophagosomal structures to persist in immature autophagosomal vacuoles for a period of time that is suitable to differentiate into an acid-resistant replicative form. Then, the subsequent secretion of the effector LepB releases the block to autophagosome maturation, and the adapted progeny continues to replicate within autophagolysosomes.

During infection, *Coxiella* is able to regulate host trafficking pathways by the selective retention of Rabs on membranes of the PV [71] (Figure 2). The small Rab GTPases regulate vesicular trafficking and autophagy [72]. In eukaryotic cells, organelle identity is determined, in part, by the presence of active Rabs on the membranes of each organelle. Some protein effectors ejected by *Coxiella* may work as specific receptors for Rabs GTPases or as enzymes that post-translationally modify Rab proteins or endosomal membrane lipids essential for Rab functions [29].

Several works have described the role and distribution of Rab GTPases during *Coxiella* infection and its relationship with autophagy. This bacterium transits through the normal endo/phagocytic pathway actively interacting with autophagosomes at early stages p.i.. *Coxiella* phagosomes acquire Rab5 and Rab7 proteins sequentially during infection, and colocalize with the marker of acidic compartments LysoTracker and the autophagy markers monodansylcadaverine (MDC) and LC3. The LC3 acquisition appeared to be a bacterially-driven process, because it is inhibited by the bacteriostatic antibiotic chloramphenicol [73]. Furthermore, the inhibition of the autophagic process with 3-methyladenine or wortmannin blocked *Coxiella* vacuole formation in HeLa cells [74]. Moreover, the overexpression of LC3 or the GTPase Rab24 in CHO cells accelerates the development of *Coxiella* vacuoles. In contrast, overexpression of mutants of those proteins that cannot be targeted to autophagosomes dramatically decrease the number and size of the vacuoles in the first hours of infection [75].

Autophagy is an essential process modulated by the T4SS during *Coxiella* infection. Via this system, the pathogen actively recruits autophagosomes to the PV to deliver nutrients and provide membrane for the expanding vacuole. Accordingly, interactions with autophagosomes are necessary for the PV maturation to a phagolysosome-like compartment. *Coxiella* effector proteins control interactions with autophagosomes in macrophages at early stages of infection; and this progression is necessary for PV fusion with lysosomes. Winchell *et al.* have demonstrated the localization of LC3 and p62 in the wild type *Coxiella* PV but their absence in T4SS mutant organism-containing phagosomes in human macrophage-like cells, primary human alveolar macrophages and CHO cells [76]. However, while the lipidated LC3 levels were elevated regardless of the T4SS activity, p62 levels remained constant during *Coxiella* growth, suggesting that the pathogen does not promote the turnover of the autophagic p62 [77]. Altogether, these evidences suggest that the transit through the autophagic pathway increases the bacterial load by providing a favorable niche for the intracellular differentiation and survival.

In addition to the interaction of *Coxiella* phagosomes with both the endocytic and the autophagic pathways, our laboratory has reported that the *Coxiella* PV also intercepts the secretory pathway. Rab1b is a small GTPase responsible for the anterograde transport between the endoplasmic reticulum and the Golgi apparatus. It has been demonstrated that Rab1b is recruited to the PV at later infection stages (6 h p.i.) and, interestingly, the knockdown of Rab1b altered the vacuole growth, indicating that this protein is required for a suitable biogenesis of the *Coxiella* PV. In addition, disruption of the secretory pathway by chemical inhibitors or by overexpression of mutant proteins, affected the typical size of the PV [78].

Due to an extended growth cycle, *Coxiella* continuously manipulates cellular processes to generate the PV and to increase host cell viability. Thus, *Coxiella* is able to modulate several host signaling pathways to ensure survival and intracellular replication [79]. In addition to the influence in autophagy, *Coxiella* also regulates apoptosis to its own benefit. These effects are achieved through the Beclin 1/Bcl-2 interplay. We have demonstrated that Beclin 1 is recruited to the *Coxiella*-membrane vacuole, favoring development and bacterial replication. In contrast, the anti-apoptotic protein Bcl-2 alters the normal development of the *Coxiella* replicative compartment, even though it is also recruited to the vacuole membrane [80]. Regarding apoptosis regulation, this pathogen is also capable of activating the pro-survival kinases Akt and Erk1/2 to increase host viability [79].

Although Rab GTPases and pathways such as autophagy and apoptosis are important targets of *Coxiella* infection, there are yet underexplored functions of bacterial effectors involved in the generation of the replicative PV.

### 2.4. Legionella Pneumophila

The severe pneumonia known as Legionnaires' disease follows the infection of the gram-negative bacterium *Legionella* *pneumophila* (hereafter *Legionella*). This pathogen can be engulfed by eukaryotic phagocytes. As an escape mechanism, *Legionella* has developed a complex system of effector proteins which allow the bacterium to survive within the phagocytic vacuole, thus escaping from the host’s defense mechanisms and replicating within a self-tailored compartment. The course of infection can be divided into five main phases: 1- pathogen uptake, 2- formation of the replication-permissive vacuole, 3- intracellular replication, 4- host cell response, and 5- bacterial exit. *Legionella* effector proteins target every stage of this process, interacting with the secretory, endosomal, lysosomal and autophagy pathways, as well as with mitochondria [81].

Similarly to *Coxiella*, one virulence strategy used by *Legionella* is to manipulate host cellular processes using bacterial proteins that are delivered into the cytosolic compartment of the host cell by the specialized T4SS [82]. The pathogen translocates more than 300 bacterial proteins, whose functions are mostly still unknown. Nevertheless, several investigations suggest their roles in the modulation of diverse host processes such as vesicle trafficking, autophagy, ubiquitination, and apoptosis [83,84]. In the next paragraphs, the *Legionella* effectors mediating functions in vesicle trafficking and autophagy are discussed.

After phagocytosis, *Legionella* actively evades endocytic trafficking and creates an ER derived niche. The biogenesis of *Legionella*-containing vacuoles and autophagosomes share several features, including the presence of ER-derived membranes [85,86,87]. However, *Legionella* has also acquired mechanisms to initially evade the autophagic pathway [88]. Within the first hours of infection, it has been demonstrated that effector proteins such as DrrA/SidM, LidA, and RalF, secreted by the T4SS, prolong the association time of the vacuole with the ER and inhibit the immediate delivery to lysosomes [85]. Moreover, it has recently been described that the effector RavZ cleaves the membrane-conjugated LC3 from autophagosomal structures [89]. Thus, bacteria persist in immature autophagosomal vacuoles for a period that is suitable for them to differentiate into an acid-resistant replicative form [90]. Then, the subsequent secretion of the effector LepB releases the block to autophagosome maturation, and the adapted progeny continues to replicate within autophagolysosomes [85]. By 18 h p.i., a significant proportion of vacuoles contain LAMP-1, cathepsin D, and acidic pH [90], and are labeled by autophagic proteins [86].

Among the diverse set of regulatory molecules affected by the T4SS effectors, small host GTPases seem to be prominent and significant targets. The master regulators of vesicular trafficking, Rab proteins, are mainly targeted by *Legionella* proteins, and among these, Rab1 undergoes the most diverse modifications [91]. *Legionella* affects the intracellular vesicular trafficking of infected eukaryotic cells by recruiting the small GTPase Rab1 to the cytosolic side of the pathogen-containing vacuole. In order to achieve this process, the *Legionella* effector DrrA contains a specific guanine nucleotide exchange activity for Rab1 activation that exchanges guanosine triphosphate (GTP) for guanosine diphosphate (GDP) on Rab1. Thus, DrrA restricts the access of GTPase activating proteins, thereby maintaining Rab1 constitutively active [92]. On the other hand, by crystal structure studies and biochemical analyses, it has been demonstrated that LepB can inactivate Rab1 by acting as a GTPase-activating protein (GAP). Surprisingly, LepB can additionally operate as a GAP for Rab3, Rab8, Rab13 and Rab35, suggesting that it has a broader cellular role than it was previously thought [93].

Additionally to the non-covalent modifications that modify the nucleotide binding state of Rab1, the bacterium also uses covalent modifications such as adenylation (AMPylation) to manipulate the dynamics of Rab1 on the *Legionella*-containing vacuole [94]. It has been described that the AMPylation of Rab1 by SidM can be reversed by the *Legionella* effector SidD [94,95], indicating that *Legionella* may also use covalent modifications in order to alter the activity of Rab proteins.

Another *Legionella* effector protein, the Lpg0393, which interacts with Rabs from the endocytic pathway, has recently been identified. Due to its similar structure to the catalytic core of Rabex-5 which activates Rab5, Rab21 and Rab22, Lpg0393 exhibits a guanine-nucleotide exchange factor activity toward endosomal Rabs [96]. Since the Rab5 sub-cluster members mediate endocytosis and endosomal maturation, and the *Legionella* vacuole gathers membrane materials from endoplasmic reticulum, the activity of these Rabs may increase vesicle traffic from endosomes to trans Golgi network and eventually to the PV.

In addition, mass spectrometry studies have evidenced the interactions of the effector PieE with multiple Rab GTPases, including Rab1a, Rab1b, Rab2a, Rab5c, Rab6a, Rab7, and Rab10. The binding of the Rab GTPases with PieE was validated by yeast two-hybrid binding assays [83]. Nevertheless, the specific role of PieE remains to be elucidated.

LidA is another effector protein ejected by the *Legionella* T4SS that interacts with several host GTPases of the Rab family, including Rab6a, a regulator of retrograde vesicle trafficking within eukaryotic cells. LidA preferentially binds Rab6a in the active GTP-bound conformation by efficiently blocking the hydrolysis of GTP, even in the presence of cellular GTPase-activating proteins [97]. The role of Rab6a for *Legionella* growth within host cells is still unclear but evidence suggests that its function must be important for efficient intracellular bacteria replication.

The small GTPase Ran has been proved to play a role in a variety of cellular processes, such as nuclear pore translocation, mitotic spindle assembly and post-mitotic nuclear envelope formation. Furthermore, Ran plays an important role in cytoplasmic events involving non-centrosomal microtubules, such as endocytic receptor trafficking and the specific retrograde signaling along microtubules in nerve axons [98]. Lately, it has been found that Ran decorates the *Legionella*-containing vacuole. In macrophages infected by *Legionella*, Ran is activated by the T4SS substrate LegG1, leading to microtubule stabilization and promoting intracellular pathogen vacuole motility and bacterial growth, as well as chemotaxis and migration of *Legionella*-infected cells [99].

*Legionella*-infected cells also exhibit resistance to apoptotic stimuli due to the presence of bacterial effector proteins. SidF is a *Legionella* effector that prevents infected cells from undergoing apoptosis and thus allows maximal bacterial multiplication. SidF contributes to apoptosis resistance by specifically interacting with and neutralizing the effects of BNIP3 and Bcl-rambo, two pro-apoptotic members of the Bcl2 protein family [84]. Therefore, the inhibition of host pro-death proteins by translocated effectors constitutes a mechanism to protect host cells from apoptosis.

*Legionella* uses hundreds of effectors in order to direct cellular processes such as membrane trafficking and innate immune responses. The rigorous spatiotemporal regulation of their functions is likely to be needed for the modulation of distinct host cell events at different phases of infection as bacterial replication and the exit from the vacuole following replication. Further work is necessary to elucidate the functional role of *Legionella* effectors to provide new concepts in host–pathogen interactions.

## 3. Conclusion

Intracellular bacterial pathogens produce a variety of human diseases and cause significant morbidity and mortality in humans. Pathogens must manipulate the host cell to obtain the nutrients required for proliferation and subsequent pathogenesis. The bacteria described in this work often employ secretion systems to manipulate host processes for replication and dissemination. Through eight types of secretion systems (Type I to Type VIII), bacteria translocate pathogenic effector proteins into the host cytosol. Translocated effectors control several infection events, including the replicative vacuole formation, apoptosis, cytokine responses, and autophagy.

Autophagy is a degradation process where cellular cargos are delivered to the lysosomes. Initially considered as a response activated under stress conditions, mainly nutrient deficit, autophagy is now known to impact diverse pathophysiological conditions like aging, neurodegeneration, inflammation and infection. Not surprisingly, many intracellular pathogens including *Listeria*, *Shigella*, *Salmonella*, *Mycobacterium* and *Staphylococcus* are known to evade autophagy. However, others such as *Helicobacter pylori*, *Coxiella* and *Legionella* have developed sophisticated strategies to survive and replicate inside the hostile environment of a lysosome-derived organelle.

We have learned that these pathogens are able to handle a wide variety of host cell processes but further studies are needed to elucidate the role of bacterial effectors in manipulating host autophagy to clarify the pathogenesis of intracellular bacterial infections.

In summary, the study of the coordinated actions of bacterial effector proteins controlling Rab functions to promote bacterial survival, and the different strategies aimed at establishing the replicative vacuoles, is a fascinating area of research. Future work in this field must continue to expand our knowledge about the molecular mechanisms driving autophagy to target bacteria and the specific host factors necessary to eliminate invading pathogens. We believe that this knowledge may facilitate the development of treatments or the prevention of infections caused by these types of pathogens through the promotion of the use of autophagy modulators as antimicrobial agents.
